# Supplementary material for: Transcriptomic Insights into Mechanisms of Early Seed Maturation in the Garden Pea (Pisum sativum L.)
Source: Cells. 2020 Mar 23;9(3):779. doi: 10.3390/cells9030779 (PMC7140803; doi:10.3390/cells9030779)

Sprint−2,10(4)

Global Summary

%DE = 0.22

genes with fdr < 0.2 = 470 (243 + / 227 −)

genes with fdr < 0.1 = 428 (222 + / 206 −)

genes with fdr < 0.05 = 413 (215 + / 198 −)

genes with fdr < 0.01 = 353 (187 + / 166 −)

genes in genesets = 0

<FC> = 0

<t−score> = 0

<p−value> = 0

<fdr> = 0.78

Global Genelist

Rank	ID	log(FC)	p−value	fdr	Metagene	Description
1	TRINITY_DN1	2.01	2e−16	2e−15	30 x 1	
2	TRINITY_DN3	1.31	2e−16	2e−15	30 x 1	
3	TRINITY_DN9	−0.64	2e−16	2e−15	30 x 30	
4	TRINITY_DN7	0.97	2e−16	2e−15	30 x 1	
5	TRINITY_DN7	−1.81	2e−16	2e−15	30 x 30	
6	TRINITY_DN5	−1.37	2e−16	2e−15	30 x 30	
7	TRINITY_DN5	−1.82	2e−16	2e−15	1 x 1	
8	TRINITY_DN5	−0.88	2e−16	2e−15	1 x 30	
9	TRINITY_DN7	−1.29	2e−16	2e−15	1 x 1	
10	TRINITY_DN5	0.66	2e−16	2e−15	30 x 4	
11	TRINITY_DN4	2.03	2e−16	2e−15	1 x 1	
12	TRINITY_DN9	3.06	2e−16	2e−15	30 x 1	
13	TRINITY_DN9	0.92	2e−16	2e−15	30 x 1	
14	TRINITY_DN9	−0.85	2e−16	2e−15	1 x 1	
15	TRINITY_DN9	0.96	2e−16	2e−15	30 x 1	
16	TRINITY_DN2	−0.78	2e−16	2e−15	1 x 30	
17	TRINITY_DN2	−0.71	2e−16	2e−15	1 x 1	
18	TRINITY_DN2	1.09	2e−16	2e−15	30 x 1	
19	TRINITY_DN2	0.97	2e−16	2e−15	30 x 1	
20	TRINITY_DN2	−0.76	2e−16	2e−15	30 x 30	

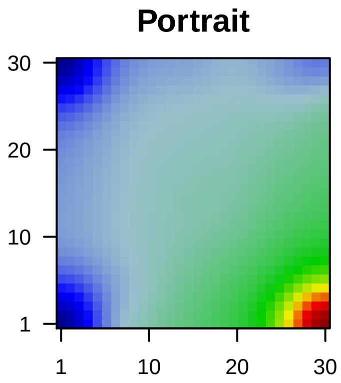

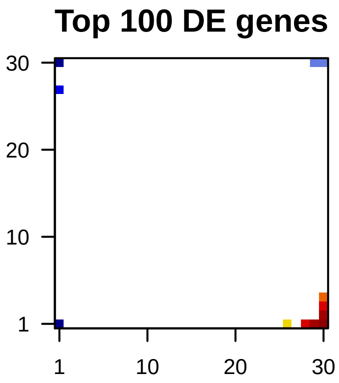

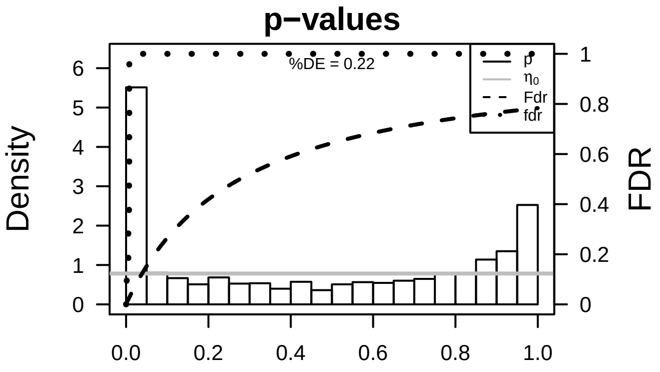

Supplement: Supplementary file 1 [file cells-09-00779-s001.zip › Supplementary materials/FileS4.zip!/FileS3/TFs - Results/Summary Sheets - Samples/Sprint.2.10.4..pdf]
